# Bioremediation of Bisphenol A and Benzophenone by Glycosylation with Immobilized Marine Microalga *Pavlova* sp.

**DOI:** 10.4137/EHI.S2758

**Published:** 2009-09-23

**Authors:** Kei Shimoda, Hiroki Hamada

**Affiliations:** 1 Department of Chemistry, Faculty of Medicine, Oita University, 1-1 Hasama-machi, Oita 879-5593, Japan; 2 Department of Life Science, Faculty of Science, Okayama University of Science, 1-1 Ridai-cho, Kita-ku, Okayama 700-0005, Japan. Email: hamada@dls.ous.ac.jp, shimoda@med.oita-u.ac.jp

**Keywords:** phytoremediation, endocrine disrupting compounds, marine microalgae, immobilized cells, glycosylation

## Abstract

Cultured cells of *Pavlova* sp. glycosylated bisphenol A to its mono-glucoside, 2-(4-β-D-glucopyranosyloxyphenyl)-2-hydroxyphenylpropane (9%). Use of immobilized *Pavlova* cells in sodium alginate gel improved yield of the product (17%). On the other hand, *Pavlova* cell cultures converted benzophenone into diphenylmethanol (49%) and diphenylmethyl β-D-glucopyranoside (6%). Incubation of benzophenone with immobilized *Pavlova* cells gave products in higher yields; the yields of diphenylmethanol and diphenylmethyl β-D-glucopyranoside were 85 and 15%, respectively.

## Introduction

Bisphenol A^1^ and benzophenone^1^,^2^ have been listed among “chemicals suspected of having endocrine disrupting effects” by the World Wildlife Fund, the National Institute of Environmental Health Sciences in the USA and the Japanese Environment Agency. These biphenyl compounds exhibited estrogenic activity in bioassays.^1^,^2^ These compounds are widely used to manufacture polyacrylates, ether resins, phenol resins, insecticides, agricultural chemicals, pharmaceuticals, and coatings, and their residues are released as pollutants into rivers and seas.^3^ In the case of bisphenol A, worldwide production capacity was estimated about 1,100 million pounds.^4^ From the viewpoint of seawater pollution control, metabolism of bisphenol A and benzophenone by marine microalgae is of importance. There have been several reports on the biodegradation of these biphenyl endocrine disrupters by bacteria so far. They are broken down by oxidative cleavage catalyzed by specific peroxidases in bacteria.^5^–^10^ Recently, it has been reported that freshwater microalgae converted bisphenol A into its mono-glucoside.^11^ However, little attention has been paid to the metabolism of bisphenol A and benzophenone by marine microalgae. Because *Pavlova* is a marine microalga generally found in a coastal lagoon, its use for bioremediation of environmental pollution is of interest.

We report here the reduction and glycosylation of bisphenol A and benzophenone by marine microalga *Pavlova* sp. Use of immobilized *Pavlova* cells improved the conversion yield of the products.

## Results and Discussion

### Biotransformation of bisphenol A

**Figure 1.**
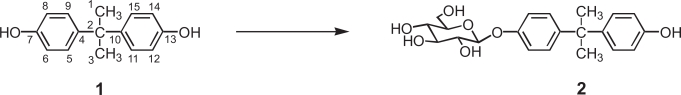
Glycosylation of bisphenol A (**1**) by free suspended and immobilized *Pavlova* cells.

The biotransformation products were isolated from *Pavlova* cell cultures, which had been incubated with bisphenol A (**1**) for five days, by a combination of Diaion HP-20 column chromatography and preparative HPLC. The structure of the product **2** was determined as 2-(4-β-D-glucopyranosyloxyphenyl)-2-hydroxyphenylpropane (9%) by FABMS, ^1^H and ^13^C NMR analyses (Fig. 1).^11^ Aromatic compounds have been reported to be degraded through the breakdown of their aromatic ring by some microorganisms.^5^–^10^ Biotransformation of bisphenol A by *Pavlova* cells is quite different from that by these microorganisms.

In our recent study, it was found that the endocrine disrupting activity of glycosyl derivative of bisphenol A decreased 80% in comparison with that of bisphenol A.^12^ The present glycosylation system using *Pavlova* cells is useful for chemical modification of the biphenyl endocrine disrupter, bisphenol A.

Next, bisphenol A (**1**) was subjected to the biotransformation with immobilized *Pavlova* cells in 2% sodium alginate gel. Use of immobilized *Pavlova* cells improved the yield of product to 17%. These findings suggested that stabilization of *Pavlova* cells by immobilization enhanced their potential to produce glycosides.

**Figure 2.**
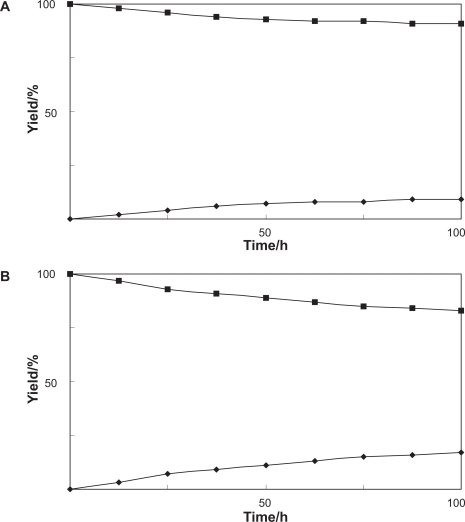
Time courses of the biotransformation of bisphenol A (**1**) by **A**) free suspended or **B**) immobilized *Pavlova* cells. Yields of **1** (▪) and **2** (♦) are plotted.

A time-course experiment was carried out to examine the ability of free suspended or immobilized *Pavlova* cells to glycosylate bisphenol A (**1**). Figure 2 (A) showed that the amount of product **2** increased with time during the reaction with normal *Pavlova* cells. On the other hand, **2** was obtained in higher yield using the immobilized *Pavlova* cells, which had been prepared at 2% sodium alginate concentration, in comparison with the case of the biotransformation by normal cells (Fig. 2B).

### Biotransformation of benzophenone

**Figure 3.**
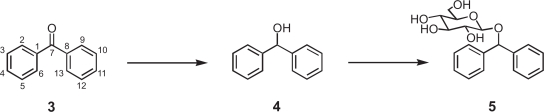
Reduction and glycosylation of benzophenone (**3**) by free suspended and immobilized *Pavlova* cells.

**Figure 4.**
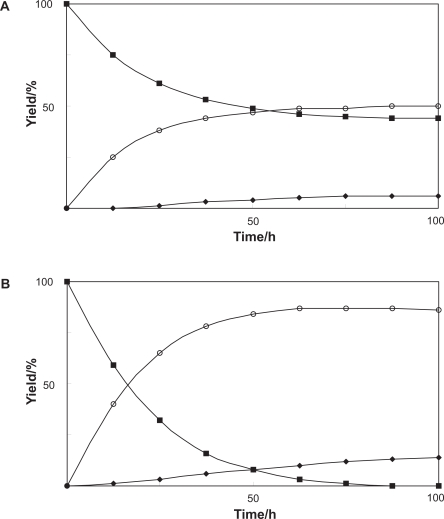
Time courses of the biotransformation of benzophenone (3) by **A**) free suspended or **B**) immobilized Pavlova cells. Yields of 3 (▪), 4 (○), and 5 (♦) are plotted.

The conversion of benzophenone (**3**) was investigated using cultured *Pavlova* cells. The products **4** and **5** were extracted and purified by the same procedures as the biotransformation of bisphenol A (**1**). The chemical structure of the products **4** and **5** was identified on the basis of their FABMS, ^1^H and ^13^C NMR, H-H COSY, C-H COSY, and HMBC spectra as diphenylmethanol (**4**, 49%) and diphenylmethyl β-D-glucopyranoside (**5**, 6%), respectively (Fig. 3). The time-course of the biotransformation of benzophenone (**3**) with cultured *Pavlova* cells showed that benzophenone (**3**) was converted into diphenylmethanol (**4**) at early stage of incubation, and that diphenylmethyl β-D-glucopyranoside (**5**) was produced after 12 h incubation (Fig. 4A). These findings indicated that formation of glucoside **5** occurred following to that of alcohol **4** as shown in Figure 3. The biotransformation system accompanied with reduction and subsequent glycosylation using *Pavlova* cells would be of use from the viewpoint of detoxification of biphenyl endocrine disrupters with no hydroxyl group but carbonyl group.

Use of immobilized *Pavlova* cells improved the yield of products; the yields of diphenylmethanol (**4**) and diphenylmethyl β-D-glucopyranoside (**5**) were 85 and 15%, respectively, after five days-incubation. Figure 4 (B) showed that the yield of two products **4** and **5** was efficiently enhanced by using immobilized *Pavlova* cells as compared with the case of the biotransformation by normal cells.

### Materials and methods

Bisphenol A (**1**) and benzophenone (**3**) were purchased from Aldrich Chemical Co. The ^1^H and ^13^C NMR, H-H COSY, C-H COSY, and HMBC spectra were recorded in CD_3_OD using a Varian XL-400 spectrometer (Varian Inc.). The chemical shifts were expressed in δ (ppm) referring to tetramethylsilane. The FABMS spectra were measured using a JEOL MStation JMS-700 spectrometer (JEOL Ltd.). HPLC was carried out on a YMC-Pack R&D ODS column (150 × 30 mm) at 25 °C [solvent: methanol-water (9:11, v/v); detection: UV (280 nm); flow rate: 1.0 ml/min]. *Pavlova*, a gift from Ehime Prefectural Fisheries Experimental Station, Japan, cells (5 g) were cultivated in a synthetic seawater (500 ml) for 2 weeks at 20 °C with constant aeration by air (1 l/min) in 1 l flasks under illumination (1000 lx). The synthetic seawater contained 20.747 g NaCl, 0.8 μg MnCl_2_·4H_2_O, 9.474 g MgCl_2_·6H_2_O, 1.326 g CaCl_2_·6H_2_O, 3.505 g Na_2_SO_4_, 597 mg KCl, 171 mg NaHCO_3_, 85 mg KBr, 34 mg Na_2_B_4_O_7_·10H_2_O, 12 mg SrCl_2_, 3 mg NaF, 1 mg LiCl, 0.07 mg KI, 0.2 μg CoCl_2_·6H_2_O, 8 μg AlCl_3_·6H_2_O, 5 μg FeCl_3_·6H_2_O, 0.2 μg Na_2_WO_4_·2H_2_O, 0.02 mg (NH_4_)_6_Mo_7_O_24_, 0.0045% Na_2_SiO_3_ and 1.07 ml of NM solution per 1 l of distilled water. The NM solution (1 l) is a kind of vitamin solutions and composed of NaNO_3_ (150 g), Na_2_HO_4_ (10 g), EDTA-2Na (0.9 g), Vitamin B_12_ (1.5 mg), thiamine·HCl (75 mg), biotin (1 mg), EDTA-Fe (2.5 g), and H_2_NC(CH_3_OH)_3_ (5 g) in distilled water.

### Biotransformation of biphenyl compounds by cultured *Pavlova* cells

Cultured *Pavlova* cells were harvested by centrifugation at 3000 rpm for 15 min and washed twice by adding 100 ml of synthetic seawater followed by centrifugation (3000 rpm for 15 min). To the 500 ml flask containing 9 g of cultured *Pavlova* cells and 300 ml of a synthetic seawater was added 0.2 mmol of substrate. The cultures were incubated at 20 °C with aerobic shaking for five days under illumination (1000 lx). After the incubation period, the cells and synthetic seawater were separated by centrifugation at 1000 g for 15 min. The synthetic seawater was extracted with ethylacetate. The cells were extracted (three times) by homogenization with methanol, and the methanol fraction was concentrated and partitioned between water and ethylacetate. The ethylacetate fractions were analyzed by HPLC, combined, and concentrated. The water fraction was applied to a Diaion HP-20 column and the column was washed with water followed by elution with methanol. The methanol eluate was subjected to preparative HPLC to give glycosylation products. The yield of the products was determined on the basis of the peak area from HPLC and expressed as a relative percentage to the total amount of the whole reaction products extracted. The control free suspended cells, which had not been treated with substrates, were subjected to the same extraction procedures and HPLC analyses as normal biotransformation experiments, and no products and substrates have been detected in the cells and in the medium despite careful HPLC analyses.

### Biotransformation of biphenyl compounds by immobilized *Pavlova* cells

Sodium alginate (2%) was suspended in water (50 ml), which was autoclaved at 120 °C for 30 min. Cultured *Pavlova* cells (9 g) were added to this solution and the mixture was stirred for 2 h until it became homogeneous. The suspension was added dropwise from a dropping funnel with a glass tube into a 5% CaCl_2_ solution (1 l) with stirring to form pieces of spherical sodium alginate gel with 5 mm diameter immediately. After washing with a synthetic seawater, immobilized *Pavlova* cells were used for biotransformation experiments.

To the immobilized *Pavlova* cells which included 9 g of *Pavlova* cells with 250 ml of the synthetic seawater in a 500 ml flask was added 0.2 mmol of substrate. The flask was incubated at 20 °C with aerobic shaking for five days under illumination (1000 lx). Isolation of products was carried out by the similar procedures to normal biotransformation experiment described above.

### Time-course experiments using cultured or immobilized *Pavlova* cells

Time-course experiments were performed by a procedure similar to normal transformation experiments.^13^–^15^ Substrate (0.2 mmol) was administered to each of eight 500 ml flasks containing 9 g of cells or immobilized *Pavlova* including 9 g cells. At a regular time interval (12.5 h), one of the flasks was tested to evaluate the yields of the products. The yield of the products was determined on the basis of the peak area from HPLC and expressed as a relative percentage to the total amount of the whole reaction products extracted.

## Conclusions

In this work, we have reported the reduction and glycosylation of bisphenol A and benzophenone by free suspended and immobilized *Pavlova* cells. The free suspended *Pavlova* cells converted bisphenol A into its glucoside product, and benzophenone into the reduction and glucosylation products. Use of the immobilized *Pavlova* cells effectively enhanced the yield of the products. The present biotransformation system including immobilized *Pavlova* cells is useful for chemical modification of these biphenyl endocrine disrupters.
